# Lowering COVID-19 vaccine hesitancy among immigrants in Norway: Opinions and suggestions by immigrants

**DOI:** 10.3389/fpubh.2022.994125

**Published:** 2022-11-16

**Authors:** Prabhjot Kour, Abdi Gele, Arild Aambø, Samera A. Qureshi, Naima Said Sheikh, Øystein Vedaa, Thor Indseth

**Affiliations:** ^1^Department of Infection Control and Preparedness, Norwegian Institute of Public Health, Oslo, Norway; ^2^Unit for Migration and Health, Department of Health Services, Norwegian Institute of Public Health, Oslo, Norway; ^3^Department of Health Promotion, Norwegian Institute of Public Health, Bergen, Norway; ^4^Department of Psychosocial Science, University of Bergen, Bergen, Norway

**Keywords:** COVID-19, immigrants, vaccine hesitancy, opinions, suggestions, Norway

## Abstract

Even though COVID-19 vaccine has been proved effective, vaccine uptake and coverage has been and still is a great concern across different immigrant groups. Vaccine hesitancy remains a barrier to accept the vaccine among immigrants across the globe—including Norway—despite higher rates of hospitalizations and deaths. This study aimed to explore the opinions and suggestions of immigrants on how to lower the COVID-19 vaccine hesitancy among immigrants in Norway. Qualitative interviews were conducted with 88 persons with different immigrant background. Data was analyzed using framework analysis, utilizing “3Cs model of vaccine hesitancy” as a theoretical framework. The analysis yielded five main themes related to factors that may lower the vaccine hesitancy among immigrants in Norway: (1) Effective cultural communication, (2) Vaccine advocacy through community engagement, (3) Motivating factors, (4) Collaborative efforts *via* government and healthcare, and (5) Incentives for vaccination. This study enhanced our understanding of factors that according to immigrants themselves may lower the vaccine hesitancy. The insights obtained in this study can contribute to a better understanding of the current status of vaccine uptake among immigrants and can further give directions on how to improve vaccine uptake in these groups in Norway.

## Introduction

In several countries, the COVID-19 pandemic has disproportionately affected immigrants due to different range of vulnerabilities, including socioeconomic barriers such as occupational exposure (bus/taxi drivers, cleaning industry etc.) with no possibility of home office, overcrowded housing, lack of or low health literacy, co-morbidities leading to higher rates of hospitalization and death in these groups, as compared to the general population in the respective countries (1–5).

The development and rolling out of COVID-19 vaccine have been a great public health achievement. High vaccination coverage induces indirect protection to the overall community or herd immunity, by decreasing the transmission rates, and thus also decreasing the risk of infection among the most susceptible and vulnerable in the community (6). However, the success of the vaccine program depends on ensuring that all members of society have equal and prompt access to the vaccine (1). Although awareness regarding COVID-19 vaccine can often be high in high income countries, it seems that many countries are still struggling to increase vaccination coverage among those who are hesitant about the vaccine (7). Vaccine hesitancy, here understood *as delay in acceptance or refusal of vaccination despite availability of vaccination services, is a complex phenomenon, dependent on the context, and associated with various social and physical factors* (8). It remains a significant challenge to public health and a barrier to succeed with the disease containment strategy (7, 9). Recent studies have reported that immigrants are more hesitant to accept the COVID-19 vaccine, in comparison to the general population (10–18). To the extent that vaccine hesitancy is reflected in vaccination coverage and uptake, the following numbers might throw some light on the problem. In Sweden, the lowest vaccination coverage was reported among immigrants born in low- or middle-income countries (North Africa: 59%, other African countries: 44%) as compared to those born in Sweden (91%) (16, 17). In the UK, despite high availability of vaccines, the proportion who have chosen not to take the vaccine was higher among Blacks (71.8%), Pakistani and Bangladeshi (42.3%) in comparison to White British (15.2%) (10). Racial disparities with COVID-19 vaccine uptake have also been reported in a study conducted in the US, in which Black Americans were least likely to accept the vaccine as compared to other groups (12).

In a survey from Norway, it was reported that COVID-19 vaccine uptake varied between different immigrant groups. Immigrants from Eastern Europe, Western Asia and Africa had significantly lower uptake than the general population. The authors linked such difference to length of residence in Norway, education levels, and contact with Norwegians (19). Further, a recent Norwegian study on COVID-19 vaccination coverage by immigrant background, reported that immigrants had lower vaccine uptake, which varied from 45% (Latvia, Bulgaria, Poland, Romania, and Lithuania) to 92 % (Vietnam, Thailand, and Sri Lanka). The authors suggested that the difference in vaccination coverage to some extent could be explained by income and education (20). In another study, it was found that vaccination coverage in European countries ranged from 24.3 to 98.1% and ranged between 44.0 and 89.2% among European-born immigrants in Norway. Higher vaccination coverage was found among immigrants with a longer stay of residence in Norway than those with a shorter stay (21). Furthermore, lower vaccination uptake was also reported among health professionals with immigrant background in Norway (22). In a nation-wide registry study among healthcare workers, the vaccination rate was 9-percentage point lower among immigrant health workers (85%) compared to healthcare workers with non-immigrant background. The overall vaccination rate varied between health workers with immigrant background. The lowest vaccination rates were found among those born in Somalia (78%), followed by Eritrea (77%), Poland (76%), Romania (75%), Lithuania (72%), Serbia (72%) and Russia (71%) (22). We assume that a great deal of these differences in vaccination coverage and uptake are due to vaccine hesitancy.

Various studies have reported reasons for vaccine hesitancy among immigrants such as in Danish study, it was pointed out that hesitancy may be caused by lack of information about vaccine due to language barriers and limited digital competencies (23). Among younger immigrants, of whom several were not afraid of getting infected with virus or already had been infected, there was a tendency to deem vaccination as an unimportant measure. Moreover, some had received misinformation of infertility as the long-term side effect (23). Similar findings were reported in studies from Sweden and the UK, in addition to having the distrust on institutions (10, 11, 24–26).

A study on the impact of vaccine misinformation in the UK and the US suggests that some migrant communities may be more susceptible to COVID-19 vaccine misinformation, particularly where language barriers and social exclusion contribute to a deficit of accurate and accessible information (27). In previous work, we have explored the barriers to COVID-19 vaccination among immigrants in Norway and found out that immigrants were hesitant to receive the vaccine because of fear of side effects, long-term complications, misinformation of vaccine contents, conspiracy theories and lack of professional guidance on vaccine safety (28).

In line with this, Njoku et al. (29) discuss various ethnic inequities and structural barriers that can lead to lower vaccine acceptance, such as immigration status, lack of a centralized system, complicated vaccine scheduling, difficulties in reaching a vaccination site, language difficulties and inaccurate translations, poor digital access, and lack of trusted point of access in immigrant-specific areas. Indeed, vaccine hesitancy is a complex concept, varying across time, place and situations. Still, because vaccination is the most promising solution for the COVID-19 pandemic, such hesitation may influense vaccine utptake, create difficulties in obtaining adequate vaccine coverage in some immigrant groups and thus pose a threat to immigrants health (29).

In the present paper, we explored immigrants' perspectives, opinions, and experiences regarding interventions with a potential to lower vaccine hesitance and thus increase the uptake of COVID-19 vaccine among immigrants in Norway. Increasing vaccine uptake among immigrants is of significant importance in Europe. However, to our knowledge, so far there has been no study in Europe that has explored immigrants' perspectives on interventions to increase their vaccine uptake.

### Theoretical framework

**Figure 1:**
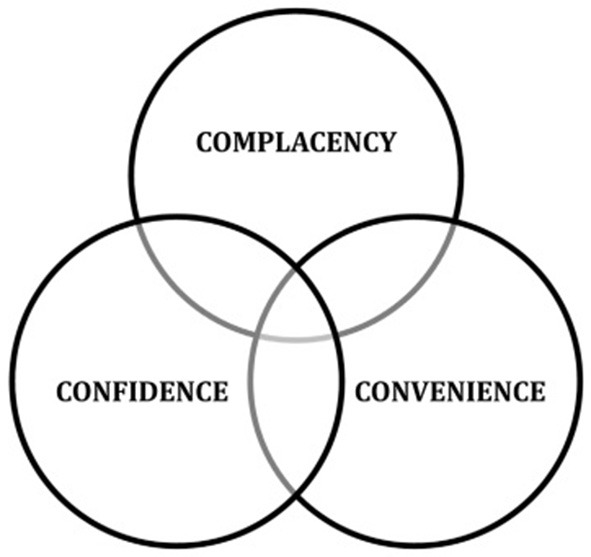
“3Cs model of vaccine hesitancy” (8).

WHO's Strategic Advisory Group of Experts (SAGE) on immunization has proposed a 3Cs-model (complacency, convenience, and confidence, Figure 1) (8) has been proposed in order to understand the concept of vaccine hesitancy and its determinants. *Complacency* occurs when the perceived risks of vaccine-preventable diseases is perceived as low. Vaccination is therefore not considered as a necessary preventive measure. In turn, complacency is influenced by factors such as health/life responsibilities. *Convenience* is another detrimental factor that affects the decision to get vaccinated. It depends on the quality of service that is made available, such as vaccine delivery at a particular time and place, appeal of immunization services, affordability, language, and health literacy, as well as the cultural context. Further, in the 3C-model, *confidence* means the ability to trust the effectiveness and safety of the vaccine, which in turn is influenced by the reliability and competence of the health care services and government's motivation for installing a vaccination program (8).

In on our study, *complacency* could be understood as “no need”, *convenience* could be referred to the contextual factors, such as appeal, affordability of services, health literacy and social support. Further, *confidence* could imply “lack of trust in vaccine and/or services.

As vaccine hesitancy results from complex decision-making processes that to varying degrees are influenced by each of the factors constituting the 3Cs model (8), we believe that these factors in many instances also can be overlapping. Indeed, the descriptions of the 3Cs above as well as Figure 1 show that these categories are not mutually exclusive. Nonetheless, we argue that the 3Cs-model can be of great value in identifying factors that may lower vaccine hesitancy. MacDonald et al. do, however, not discuss the overlap among these 3Cs in their model.

## Methods

To gain knowledge about factors that can help in lowering the vaccine hesitancy among immigrants, we conducted a qualitative exploratory study.

### Recruitment and participants

The study was conducted in several counties of Norway and included those who met the inclusion criteria: men and women, over the age of 18 and who were either born outside of Norway [immigrant (30)] or had at least one parent born outside of Norway [Norwegian born to immigrant parents (30)].

To recruit participants, the Norwegian Institute of Public Health (NIPH) hired Opinion, a research consultancy firm. Out of the 55 participants recruited by this firm, 28 were recruited by snowballing and *via* networks like Facebook and other social media. In addition, five participants were recruited *via* contacts in various organizations. Three informants were recruited by the moderator on the streets in Oslo. The other 34 participants were recruited by the researchers from NIPH using purposive and snowballing methods. Saturation was achieved when no new patterns, ideas and opinions were found during the course of last interviews (31).

To attain the information-rich data, the study included a diverse sample of immigrant groups, including those groups who had higher hospitalizations and mortality due to COVID-19 disease as compared to other groups and the general population (2).

A total of 89 participants (Table 1), from ten different countries, were included in the study. These were Afghanistan (12), Bosnia/Serbia (6), Eritrea (6), Iraq (12), Pakistan (12), Poland (11), Somalia (12), Sri Lanka (6), Syria (6), Turkey (6). The age of participants varied from 19 to 78 years. There were 39 men and 50 women. The length of residence among participants varied between 1 and 48 years and a few were born in Norway. Several of the participants had completed secondary education while some had university education. They worked in different sectors like health services, transport, kindergartens, restaurants, IT, education sector or they ran their own businesses. However, some of the participants were laid off from their jobs due to the pandemic and a few were students and pensioners. Some of the participants were engaged in voluntary work in their local communities and had contributed to the dissemination of information about the pandemic in their respective communities. Further, the knowledge of Norwegian society and language skills varied among the participants, according to the length of residence in Norway.

**Table 1:** Characteristics of study participants.

**Variables**	**Frequency**
	**(*n* = 89)**
**Gender**
Male	39
Female	50
**Age, years**
19–50	69
51–78	20
**Country of origin**
Afghanistan	12
Bosnia/Serbia	6
Eritrea	6
Iraq	12
Pakistan	12
Poland	11
Somalia	12
Sri Lanka	6
Syria	6
Turkey	6

### Data collection

Six researchers from NIPH conducted 34 interviews. Immigrants from Pakistan, Somalia and Iraq were interviewed in their mother tongue (by researchers with Pakistani, Somali, and Iraqi background, while those from Poland, Afghanistan and Eritrea were interviewed in Norwegian. One Iraqi participant was interviewed in Norwegian on his preference.

Two moderators from Opinion conducted 54 interviews, 53 were individual interviews (31), while one was dyadic interview (conducted with two participants) (32). Five interviews were conducted in the participant's mother tongue using interpreters, 2 were conducted in English and the remaining were conducted in Norwegian. All interviews took place during the ongoing COVID-19 pandemic in Norway, and after the roll out of a vaccination program, between March-May 2021.

The interviews were conducted using an interview guide prepared by number of researchers working in the field of migration and health at NIPH and Opinion. The guide consisted of open-ended questions, and covered the broad topics, including, knowledge about vaccines, concerns and attitudes about vaccine in their community, opinions and suggestions on what would encourage the people of their community to get vaccinated, and what according to them can help lowering vaccine hesitancy among their community members, and were followed by probing questions.

The interviews, which lasted between 30 and 90 min, were conducted digitally *via* Zoom or Teams or by telephone. One dyadic interview was conducted face to face. All the interviews were audio recorded.

### Ethical considerations

All the participants were sent information about the study prior to their participation. Prior to each interview, information regarding the study and the purpose of the study was explained to participants and consent was sought orally (a professional interpreter was used when necessary). The Regional Ethics Committee for Medical and Health Research in Norway has assessed that the topics investigated in this research project fall outside the Health Research Act. The research project therefore does not have an ethical approval from the Regional Ethics Committee in Norway. However, the ethical aspects of the current research project have been assessed by NIPH and found to be acceptable. In consultation with the Privacy Ombudsman at NIPH and Opinion, we have also assessed whether the data collection in this study required a complete Data Protection Impact Assessment (DPIA), of which it was concluded that this was not necessary.

### Data analysis

All the interviews were transcribed verbatim into Norwegian by two moderators from Opinion and five researchers from NIPH. The data analysis was done manually by framework analysis (33) using “3Cs model of vaccine hesitancy” (8) (Figure 1) as a framework.

**Figure 2:**
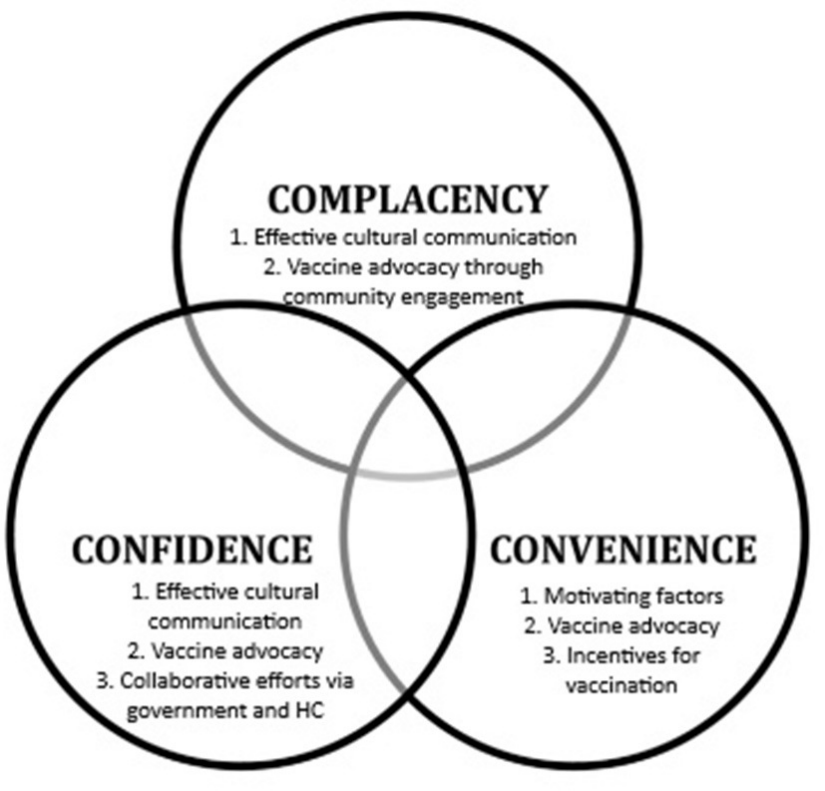
Factors lowering vaccine hesitancy among immigrants. Figure adapted from “3Cs model of vaccine hesitancy” (8).

The analysis process included five steps (33). In the first step, familiarization, the transcriptions were read and reread to become familiar with the material, to get an overview and become aware of recurring themes. In step two, five main themes were generated in dialogue with the original “3Cs model of vaccine hesitancy”. That is, the themes were generated from the data considering the theoretical framework as a correspondence. In the third step, indexing, data corresponding to a particular theme was identified and pooled. In step four, charting, the indexed data from stage three were placed in the thematic framework. The themes were discussed by the researchers among themselves several times, addressing the credibility of the findings. In the final stage of mapping and interpretation, the key themes in the thematic framework were described and interpreted in the results section. Two of the themes overlapped within the three categories of the 3Cs model (complacency, confidence, and convenience), as shown in Figure 2. This overlap is explained and discussed in the discussion section.

## Results

The analysis yielded five main themes that represented factors which may lower the vaccine hesitancy among immigrants in Norway. These themes were: (1) Effective cultural communication, (2) Vaccine advocacy through community engagement, (3) Motivating factors, (4) Collaborative efforts *via* government and healthcare (HC), and (5) Incentives for vaccination. Themes 1 and 2 were overlapping in the framework of “3Cs model of vaccine hesitancy” (Figure 2), and also formed the basis for our discussion.

### Effective cultural communication

Participants mentioned that vaccine hesitancy and skepticism can be lowered by effective communication among different immigrant groups in their respective languages. They further stated that addressing the medical and religious concerns of these groups along with targeting false information on social media will create awareness of vaccine safety. They also suggested involvement of public figures and leaders in campaigning for vaccine safety, especially people who have been vaccinated and are trusted in the Norwegian society.

*In Skien, every person who gets vaccinated, makes a video in their native language to recommend the vaccine. We can use these videos in social media and suggest people to take vaccine*. (F, 50 years)

*I think it is important that it comes from a local network, including someone who speaks the language. I think it is important to have an open dialogue about these conspiracy theories and perceptions, “Why are you sceptical of the vaccines? What is the cause? Do you have any reason?* ≫ *I do not think it should be downplayed, then, that people feel that unrest. That unrest exists among both the younger and older generations, but for some it may simply be a lack of knowledge. So, I think it's important to use local people and also translate content about what this vaccine means and have open discussions about it… that you could, for example, have a workshop or something where you talk openly about what the vaccine means and why you are so afraid to take it* (F, 29 years)

…* when it comes to vaccination, I think that the communication could have been better adapted to culture* (M, 29 years)

Participants suggested that providing detailed information about vaccine, that is, the benefits of receiving the vaccine in their own language and free of cost availability will be beneficial, especially if it gets circulated within their local networks and places where they often meet. The participants also mentioned targeting false information by having an open dialogue with group of immigrants about the potential perceptions related to concerns and conspiracy theories.

*I think people need more detailed information about the vaccine. Most people do not believe in vaccine, and it is important that they are informed about it. Now we have made brochures and hung them up in different places where people often meet*. (M, in his 30s)

…* for very many it is not enough to say that the vaccine is safe. They feel that they are not treated well, when they are told that, just like children, you see. They want accurate detailed information, not just the general “vaccine is safe”*. (F, 33 years)

Some participants raised the concern about long history of skepticism related to any vaccine in their home country, which ultimately influence their decision making. They suggested to target these concerns and assure people of their community that vaccine is safe, by providing examples in their language, even if the vaccine for COVID-19 disease is made in short span of time.

*There has been skepticism about the vaccines in Poland for many years. This applies not only to the corona vaccine, but also to other vaccines, so it is in a way an extension of what has been already there, and at the same time so many things happened in a short time. So, people are starting to get confused. This should be addressed*. (M, 37 years)

### Vaccine advocacy through community engagement

Some participants suggested vaccine advocacy through engagement of community leaders, for example by including imams or other religious leaders in creating awareness about the benefits of receiving vaccine. This will counter the skepticism of many individuals who follow imams. Some participants also suggested about sharing their positive experiences of taking other vaccines in their home countries. This will indirectly increase the trust in COVID-19 vaccine and its safety. Further, one participant mentioned about sharing the information about vaccine from the governmental websites to their local Facebook groups to create awareness about vaccine and thereby lowering vaccine hesitancy.

*I have also shared information regarding vaccination during Ramadan from Oslo municipality page on Facebook because there are many who wonder about this. In addition, I have asked two Afghan imams for their opinions regarding vaccination when fasting, and they both said that “it goes perfectly well, not only vaccination, but also that you can take other medicines”. It is very important to convey such information about vaccine because there are still many who are skeptical to vaccine*. (M, 40 years)

As per several participants, trust building is an important factor for the success of vaccination program among immigrant communities. They mentioned that health professionals, especially those with a similar background and who speaks the same language, play a crucial role in creating trust, for example by providing adequate and clear medical advice regarding vaccination. One participant mentioned that immigrants do trust advice of health professional with similar background.

*I would not say it is safe or unsafe if the health professionals say it is safe and I will listen to them. What gave me trust and assurance was when health professionals found out that Johnson and AstraZeneca were not completely safe, and they stopped it. It made me feel even safer… and I have no problems in being vaccinated”* (M, 58 years).

### Motivating factors

Participants mentioned several motivating factors that can help in lowering vaccine hesitancy, including receiving detailed information about vaccine, information about others who have already been vaccinated and the people they trust, also that vaccination is available free of cost for everyone in the community, and that getting vaccinated will contribute to normalizing day to day life (pre-COVID-19).

Some participants mentioned that people in their communities will feel reassured if someone with a similar background has taken the vaccine, and without negative consequences, provided that the source of information is reliable. This will also contribute to the spread of a positive message about vaccine within their communities and hence lower the vaccine hesitancy.

*I have experienced that good news or medical advice spreads faster in the local community. If you know someone who has taken the vaccine and they are having that conversation with you, it will be more natural to hear them than someone from the authorities. So, if I was able to vaccinate people with minority backgrounds then I would have started with those who have the most contact with others because then they can tell and spread it further. It is reassuring to see a person who has similar background as you and speaks the same language as you, who have taken the vaccine and says that it has gone well*. (F, 20 years)

Another participant mentioned that the spread of information that vaccination will take us a long step toward an everyday life like before the pandemic will be a motivating factor for receiving the vaccine and lowering the hesitancy.

*I think if the vaccine is effective and contributes to everyday life being normalized again, then I think it is good to get vaccinated* (F, in her 30s).

Another motivating factor was that either they themselves have worked in the healthcare or they have family members or friends who have worked in the healthcare services for a long time. This may allow easy access to the information that can counter vaccine related concerns and doubts. A few participants also mentioned that having an underlying disease can be motivating factor to take the vaccine, in order to prevent getting seriously ill with COVID-19 disease.

### Collaborative efforts *via* government and healthcare

Some participants suggested that the Norwegian government and healthcare authorities should work together against the spread of misinformation that creates vaccine hesitancy by providing sufficient and adapted information tailored to the different immigrant groups.

The participants also stated that it is important that government and healthcare authorities work together in disseminating information about vaccine safety in different immigrant communities. If they together promote the vaccine, most people in their community will take it, they said.

Trust in the government as well as trust in the health professional's assessment increases the likelihood of success of vaccination programs among immigrants. Some participants, especially those with longer stay of residence, told that they do have high level of trust in both government and healthcare, because of good previous experiences. They further mentioned that their trust level increased when the government and healthcare authorities decided to stop giving AstraZeneca after it was clear that it could cause severe side-effects.

*I trust the Norwegian government, and I am sure that they will not allow the vaccine without knowing the usefulness and effect of the vaccine. For example. The blood clots that came from the AstraZeneca vaccine caused people to fear and worry about the vaccine. But the good thing was that they stopped the vaccine immediately and continued with other safe vaccines*. (M, 35 years)

### Incentives for vaccination

Several participants mentioned that they during the pandemic have taken or would take vaccine in order to travel, especially to their home countries. Some participants mentioned that they partly live in Norway, partly in their homelands. Their strong connection with their homelands and the possibility of being able to visit their family if vaccinated would act as an incentive for vaccination.

*I have already received the first dose, and everyone I know I want to get vaccinated. Because we hope that when we are vaccinated it will be possible … to travel to our country and easier to prevent quarantine or be in shorter quarantine*. (F, 45 years)

For several of our participants, prospects of traveling would be an incentive for vaccination. Other incentives were access to restaurants, cinemas, and museums, which all during the pandemic required vaccine certificate for entry. Such incentives may increase the vaccination rates and hence indirectly reduce vaccine hesitancy.

*You cannot travel without a vaccine and since they like to travel, they will have to get vaccinated*. (M in his 40s)

## Discussion

There is substantial evidence that among immigrants, COVID-19 vaccine hesitancy is high (11, 12, 14, 15, 29). Moreover, there has been a substantial increase in the misinformation about COVID-19 vaccination, especially among immigrants (34). This seems to hold true for immigrants in Norway as well (28).

The aim of this study was to explore immigrants' perspectives, opinions, and experiences regarding interventions with a potential to lower vaccine hesitance and thus increase the uptake of COVID-19 vaccine among immigrants in Norway. As a framework to our analysis we used the “3Cs model of vaccine hesitancy” (8) and five main theme were yielded: effective cultural communication, vaccine advocacy through community engagement, motivating factors, collaborative efforts *via* government and health and incentives for vaccination.

In broad terms, our study shows that to lower hesitancy and in this way enhance uptake, people at risk (immigrants) should feel that they need vaccine. Through adequate information, people may understand that they need vaccine to reduce their risk of severe illness. Adequate information may be provided through effective cultural communication, vaccine advocacy through community engagement and motivating factors.

Our findings also show that people at risk should trust the people providing information. It is then more likely that they will take the given information seriously and make an effort to become vaccinated. These factors might increase when engaging in collaboration, that is, by vaccine advocacy through community engagement and collaborative efforts *via* government and healthcare, provided that the people at risk do not feel exploited or serve as a kind of alibi.

Further, our study shows that the contextual factors are favorable for lowering vaccine hesitancy. Ap**p**eal, health literacy and social support may increase when people are collaborating as people then get to know each other, exchange views and experiences and can support each other in making decisions, that is, through effective cultural communication, vaccine advocacy through community engagement and collaborative efforts *via* government and healthcare. These findings are all in line with the 3Cs model (8).

Furthermore, the themes overlapped within the framework model. Overlap can be explained as such, vaccine advocacy through community engagement may have effect on all Cs of the framework model. That is, vaccine advocacy may increase the “confidence” and “convenience” and decrease the “complacency”. Furthermore, effective cultural communication may have effect on two categories of the model, that is, may increase the “confidence” and decrease the “complacency”, thereby lowering the vaccine hesitancy'. These are discussed below.

It is essential that information related to vaccine is both culturally tailored and provided in different languages (1). This is elaborated in a study conducted in the UK: to reach specific target groups, it is necessary to present detailed information on side-effects and contraindications not only in multiple languages, but in a culturally appropriate and understandable manner (25), which is in line with our findings. Further, Deal et al. (25) suggested that the information must be in such a way that it does not stigmatize any community, because stigmatization could have a negative impact on trust and engagement. Still, such efforts should go along with specific campaigns to counter misinformation. These strategies may help in decreasing the “complacency” and increasing the “confidence” in the 3Cs model.

Further, adequate information is likely to both increase “confidence” and decrease “complacency” in the framework model and have a positive effect on vaccine hesitancy. Regarding contextual factors, we should, however, be aware of historical oppression as well as current disparities in care that have been linked to the mistrust in the healthcare system among immigrants (35). This resonates with a Norwegian study, which found that many immigrants mostly listen to others who speak their mother tongue and, further, prefer to listen to people in their own environment rather than to representatives of local or national authorities (36). Therefore, campaigns specifically targeted at gaining trust among immigrants may increase the likelihood of receiving the vaccine.

Collaborative efforts between government, healthcare workers and representatives from the targeted local community may increase the “confidence” in the framework model as people who deliver the vaccine are more likely to be considered reliable and competent by the group in question. These efforts may include the implementing participants' suggestions as a part of a formal Tailoring Immunization Program (TIP) approach. TIP approach has been proven successful previously and has improved the immunization programs by understanding the perspectives of the low-coverage population in Europe (37). Moreover, in our study, the participants strongly highlighted a need for effective and culturally sensitive communication. They stated that healthcare professionals with a similar background, religious leaders, or other spokespersons from the inside of closed environments can most effectively provide information that can influence vaccine hesitancy. Moreover, some immigrant groups are strongly represented in the labor market, and it is likely that encouraging employers to motivate their employees to take vaccine and spread information about the vaccine safety in their respective milieus, may also aid in lowering vaccine hesitancy as many have a trustful relationship to their employers and therefore listen to their advice. However, such an initiative poses some ethical dilemmas that should be thoroughly investigated before this becomes a recommended strategy to increase vaccine uptake.

Our study showed that longer the stay of residence in Norway, the higher is the trust in the healthcare sector. Therefore, involving immigrants with longer stay of residence and those who are active in the community could be useful in the co-production of tailored interventions (to lower vaccine hesitancy and increase uptake) and in the dissemination of such interventions. Our participants suggested to involve trusted groups or community members to advocate the vaccine's safety, as they can widely promote relevant information in their own communities. They can also be useful for those designing vaccine advocacy campaigns. This is in line with other studies, which show that local community champions can act as information point both for their own community and for those designing tailored vaccine advocacy campaigns (1, 25).

Our analysis shows that vaccine advocacy through involving representatives from local communities has an effect on all Cs of the framework model. This is in line with a recent systematic review found that advocacy through community outreach programs and educational campaigns are promising strategies for improving vaccine uptake among immigrants in Europe (38). Furthermore, this kind of vaccine advocacy, which is ultimately based on adequate and appropriate information provided by highly trusted people, may increase not only “confidence”, but also “convenience” as these people may be easy to reach and speak a language that people can easily understand. “complacency” is also likely to decrease, provided the champions adequately and correctly inform about the health hazards of COVID-19 infections.

Regarding motivating factors, some participants stated that knowing about vaccine safety and its effectiveness on reducing the spread of COVID-19 disease can increase the uptake. Others reported that spread of information *via* word-of-mouth and social media by individuals who have already taken the vaccine would lower the vaccine hesitancy. In line with other studies (25) motivating factors seem to increase “convenience” in the framework model by aiding the ability to understand the effectiveness of vaccine, and thus lower vaccine hesitancy.

## Conclusion

This study provides some insights into immigrants' experiences, opinions, and suggestions on how vaccine hesitancy with regards to COVID-19 vaccine can be lowered in their respective groups. To our knowledge, this is the first study to explore these issues in a Norwegian context. Adequate knowledge provided in an effective and culturally sensitive way, combined with vaccine advocacy through community engagement may be important factors in creating disease awareness and lowering COVID-19 vaccine hesitancy. Further, there is need for establishing cooperation with religious leaders and trusted representatives and/or resource persons from the different affected communities in order to effectively reach out to the most vulnerable. This is especially important when it comes to counteracting misunderstandings and misinformation.

Our results, which are based on participants' subjective opinions and experiences, provides nuanced data specific to the immigrant population in Norway and can therefore be useful when designing approaches to lower vaccine hesitancy in these specific populations. In addition, our findings largely correspond to findings in other, international studies and thus contribute to the broader literature on how to address and allow for some general recommendations. When designing information campaigns on sensitive and critical issues like the COVID-19 pandemic, we strongly recommend a collaborative approach, a collaboration between local healthcare professionals and government officials pervaded by a direct dialogue between them and the targeted local communities; a dialogue in which all parties are listened to and willing to adjust to each other. In this way, specific concerns can be addressed, and dissemination of sufficient information is ensured so that informed decisions about COVID-19 vaccine can be made.

## Data availability statement

The datasets presented in this article are not readily available because due to the nature of this research, participants of this study did not agree for their data to be shared publicly, so supporting data is not available. However, raw data will be made available on reasonable request to researchers from accredited research institutions, with any data that may risk loss of confidentiality redacted. Access to data will be limited to investigators who provide a methodologically sound proposal. To ensure compliance with the General Data Protection Regulation, data processing must be covered by the European Commission's standard contractual clauses for the transfer of personal data, which must be signed by the data requesters. Proposals and requests for data access should be directed to the corresponding author. Requests to access the datasets should be directed to PK, prabhjot.kour@fhi.no.

## Ethics statement

The studies involving human participants were reviewed and approved by Privacy Ombudsman at Norwegian Institute of Public Health and Opinion. Written informed consent for participation was not required for this study in accordance with the national legislation and the institutional requirements.

## Author contributions

PK and TI contributed to conception and design of the study. PK carried out the data analysis, interpretation, and wrote the first draft of the manuscript. AG, AA, SQ, NS, ØV, and TI contributed to the further analysis and the write up of manuscript. All the authors contributed to manuscript revision, read and approved the submitted version.

## Funding

The study was funded by Norwegian Ministry of Integration and Education.

## Conflict of interest

The authors declare that the research was conducted in the absence of any commercial or financial relationships that could be construed as a potential conflict of interest.

## Publisher's note

All claims expressed in this article are solely those of the authors and do not necessarily represent those of their affiliated organizations, or those of the publisher, the editors and the reviewers. Any product that may be evaluated in this article, or claim that may be made by its manufacturer, is not guaranteed or endorsed by the publisher.
